# Advances in the diagnosis, evaluation, and management of leptomeningeal disease

**DOI:** 10.1093/noajnl/vdab108

**Published:** 2021-11-27

**Authors:** Ugur Sener, Priya Kumthekar, Adrienne Boire

**Affiliations:** 1 Department of Neurology, West Virginia University School of Medicine, Morgantown, West Virginia, USA; 2 Department of Neurology, Malnati Brain Tumor Institute at the Robert H. Lurie Comprehensive Cancer Center, Northwestern University Feinberg School of Medicine, Chicago, Illinois, USA; 3 Human Oncology and Pathogenesis Program, Memorial Sloan Kettering Cancer Center, New York, New York, USA; 4 Brain Tumor Center, Memorial Sloan Kettering Cancer Center, New York, New York, USA; 5 Department of Neurology, Memorial Sloan Kettering Cancer Center, New York, New York, USA

**Keywords:** cell-free tumor DNA, circulating tumor cells, intrathecal chemotherapy, leptomeningeal metastasis, targeted therapy

## Abstract

Leptomeningeal metastasis (LM) is a devastating complication of cancer with variable clinical presentation and limited benefit from existing treatment options. In this review, we discuss advances in LM diagnostics and therapeutics with the potential to reverse this grim course. Emerging cerebrospinal fluid circulating tumor cell and cell-free tumor DNA analysis technologies will improve diagnosis of LM, while providing crucial genetic information, capturing tumor heterogeneity, and quantifying disease burden. Circulating tumor cells and cell-free tumor DNA have utility as biomarkers to track disease progression and treatment response. Treatment options for LM include ventriculoperitoneal shunting for symptomatic relief, radiation therapy including whole-brain radiation and focal radiation for bulky leptomeningeal involvement, and systemic and intrathecal medical therapies, including targeted and immunotherapies based on tumor mutational profiling. While existing treatments for LM have limited efficacy, recent advances in liquid biopsy together with increasing availability of targeted treatments will lead to rational multimodal individualized treatments and improved patient outcomes.

The tissues surrounding the brain and the spinal cord, or meninges, are comprised of the outer pachymeninges and the inner leptomeninges.^1^ The pachymeninges consist of the osteal and meningeal layers of the dura mater. The pachymeninges are served by the systemic circulation and lie outside the blood-brain and blood-cerebrospinal fluid (CSF) barrier systems. The leptomeninges include the multilayered arachnoid membranes and pia mater and contain the circulating CSF.^1^ The leptomeninges reside behind the blood-CSF-barrier and may host a number of neurologic conditions including infections, inflammatory conditions as well as cancer.^2^ Spread of cancer cells into pia mater and the arachnoid membrane is referred to as leptomeningeal metastasis (LM).

Reflecting the diffuse nature of the disease location, LM is associated with a myriad of clinical manifestations that lead to substantial morbidity. CNS involvement from LM can result in encephalopathy, headache, seizures, and multiple cranial neuropathies with associated diplopia, dysphagia, and dysarthria.^3^ Impaired CSF resorption due to LM can cause obstructive hydrocephalus and symptoms of increased intracranial pressure. Spine involvement from LM may lead to pain, weakness, dysautonomia, and genitourinary dysfunction.^3^ Despite debilitating clinical symptoms, establishing the diagnosis can be challenging due to significant limitations in available radiographic and laboratory studies.^4^

LM carries a grim prognosis. Survival following diagnosis of LM from solid organ tumors such as lung cancer and breast cancer averages less than three to six months despite treatments including supportive care, radiation therapy, and intrathecal chemotherapy.^5-8^ Some variability in prognosis based on tumor type has been reported with LM secondary to lung cancer associated with poorer prognosis compared to breast cancer and melanoma.^4-7^ Tumor subtype can influence the prognosis as well with HER2+ and hormone receptor-positive breast cancer associated with better prognosis compared to hormone negative tumors^8^ and prolonged survival reported in EGFR mutant lung cancer treated with EGFR tyrosine kinase inhibitors compared to LM from other non-small cell lung cancers (NSCLCs).^9^ This bleak outcome is a direct result of major roadblocks in the study of LM, which impede the development of effective treatments and include: 1. Diagnostic challenge; 2. Inability to quantitate disease burden; 3. Genetic heterogeneity. However, many of these barriers will soon be overcome. Herein we review these emerging diagnostic tools for better detection, quantification, and prognostication of LM as well as multiple treatment modalities that portend improvements in management of this devastating complication of cancer.

## Diagnostic Advances in Leptomeningeal Metastasis

**Figure 1.**
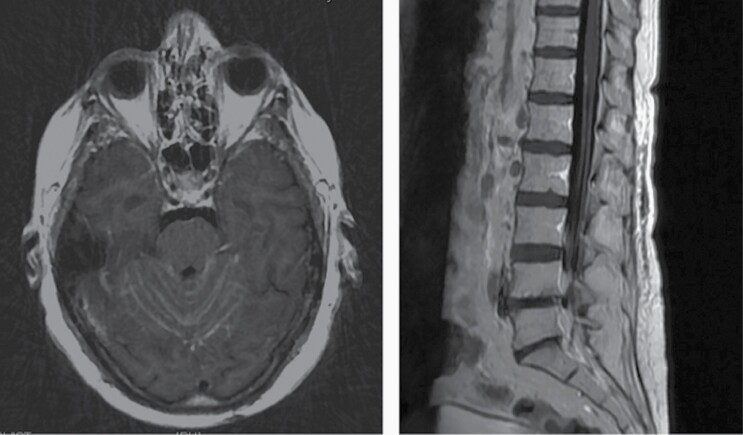
Radiographic appearance of leptomeningeal metastasis. Fifty-five year old RH woman with BRCA1 germline mutation and new onset ataxia.

Classically, LM is diagnosed on the basis of magnetic resonance imaging (MRI) and CSF cytology. Cranial LM involvement can be identified on MRI as contrast enhancement along cranial nerves and the leptomeninges, particularly in the cerebellar folia (Figure 1).^10^ LM within the spinal cord can be identified as enhancement along the spinal cord or clumping enhancement of the cauda equine.^10^ However, MRI imaging has significant limitations. Radiographic findings may not correlate with symptoms, may be difficult to distinguish pachymeningeal from leptomeningeal disease, and a normal craniospinal MRI does not exclude a diagnosis of LM.^11^ In addition, leptomeningeal enhancement noted on MRI is nonspecific and carries a broad differential including inflammatory conditions such as neurosarcoidosis and infections such as human T-lymphotropic virus.^2^

CSF profile is often abnormal in LM with lymphocytic pleocytosis, elevated protein, and decreased glucose representing the most frequently observed, but nonspecific findings.^4^ CSF cytology allows for direct visualization and identification of neoplastic cells, representing an important tool for diagnosing LM. However, sensitivity from a single CSF sample is low.^10^ Results of CSF cytology can be inconclusive, with occasional isolation of “atypical” or “suspicious” cells that are not definitively neoplastic.^11^ Multiple CSF samples may be required to improve yield with sensitivity increasing from 60% on first lumbar puncture to 85% and 90% on second and third CSF cytology analyses respectively.^10,11^

False-negative radiographic imaging and cytology analysis can lead to additional invasive testing such as repeated CSF sampling or leptomeningeal biopsy while delaying diagnosis and treatment. In addition, neither neuroaxis imaging nor CSF cytology can provide information about tumor molecular genetics. Knowledge of the molecular makeup of malignant cells in the leptomeninges is indispensable in the era of emerging targeted treatments. Emerging technologies utilizing analysis of CSF circulating tumor cells (CTCs) and cell-free tumor DNA (ctDNA) provide an opportunity for tumor analysis via liquid biopsies (Table 1).

**Table 1.** Comparison of Cerebrospinal Analysis Techniques for Detection and Characterization of Leptomeningeal Disease

	Conventional Cytology	CTC Analysis	ctDNA Analysis
Method	Direct visualization and identification of neoplastic cells	Detection of tumor cells in CSF based on surface molecule expression	Analysis of DNA released from tumor cells within CSF
Sensitivity	44–67% from first lumbar puncture, increased with serial testing	76–100% reported sensitivity for EpCAM-based detection	Not applicable
Availability	Wide-spread, available for all tumor types	Limited, requires use of specialized equipment and personnel.	Limited
Diagnostic Interpretation	Qualitative, can be inconclusive requiring serial lumbar punctures to establish diagnosis	Quantitative, but dependent on surface molecule expression	Not applicable
Biomarker potential	Not applicable	Potential use for monitoring treatment response and prognostication	Potential use for detecting resistance mutations or progression
Molecular genetics	Provides no information on tumor genetics	May be combined with downstream genetic testing to identify mutations and CSF clonal divergence	Can identify driver mutations and CSF clonal divergence

CTCs, circulating tumor cells; ctDNA, cell-free tumor DNA; CSF, cerebrospinal fluid; LM, leptomeningeal metastasis.

### Circulating Tumor Cells

Commercially available assays are available for the detection of CTCs in CSF. Originally designed for identification of CTCs in peripheral blood, one commercial assay is based on the expression of epithelial cell adhesion molecule (EpCAM), a transmembrane glycoprotein detected in cells of epithelial origin.^12^ The system for peripheral blood has been adapted to analyze CSF and detect LM from breast cancer as well as lung cancer.^12–14^ Reported sensitivities of EpCAM-based LM detection are between 76–100%, compared to 44–67% from cytology analysis on the first lumbar puncture.^12,15^ Analysis can be performed using 3 mL of CSF with ≥1 CSF-CTC/mL suggested as the optimal cutoff for diagnosis of LM.^16^ Though these small studies focused primarily on lung and breast tumors, the high reported sensitivities suggest that CTC enumeration may represent a reliable tool for detection of LM; further validation is needed and this methodology can be prone to sampling error similar to conventional cytology. EpCAM is not usually expressed by nonepithelial malignancies. In the case of melanoma, High-Molecular Weight-Melanoma-Associated Antigen/Melanoma-associated Chondroitin Sulfate Proteoglycan (HMW-MAA/MCSP) has been identified and can be used for CTC isolation.^17^ Mesenchymal tumors and tumors that have undergone epithelial to mesenchymal transition are likewise not detected by EpCAM-based assays.^17^ Further modifications to existing technology for isolation of CTCs from other tumor types will be needed. Other platforms may assist in detection of nonepithelial metastatic cells as well as cells that have undergone epithelial to mesenchymal transition.^18^

Flow Cytometry (sometimes described as “immunoflowcytometry”) may also be used to identify CTCs in CSF. In flow cytometry, CTCs are identified and quantified using fluorescently labeled antibodies against proteins such as EpCAM or HMW-MAA/MCSP.^15^ Antibodies against other tumor or organ-specific markers may also be employed in CSF analysis.^19^ One advantage of flow cytometry is that it can be performed using standard flow cytometry equipment already available in many clinical laboratories. However, standardization of detection methodology is needed to ensure results are reproducible and reliable across institutions. Moreover, while flow cytometry represents a standard diagnostic tool for the detection of LM in hematological malignancies, this technology has been only minimally employed in solid tumor LM.

Given the current limitations of both commercial EpCAM-based assays and flow cytometry methodologies, microfluidics is emerging as an alternative means of CTC isolation, with the goal of increasing sensitivity and enhancing cell recovery with potential for high-throughput processing and automation.^20^ Label-free methods based on physical characteristics of CTCs for isolation of more heterogeneous CTC samples and label-based methods leveraging affinity between CTCs and ligands are under investigation.^20^ Micropores, micropillar arrays, and optical methods are among the techniques that have been used for CTC isolation from small fluid samples based on cell physical properties such as size, density, and dielectric properties.^20^ Though these technologies are not yet widely available for CSF analysis, single cells from CSF of patients with NSCLC-associated LM have been reported.^21^ With further advances in isolation methods, incorporation of CSF CTC detection into the standard workup for LM may improve diagnostic yield, leading to earlier detection without the need for multiple conventional CSF cytology analyses and earlier initiation of treatment.

Beyond diagnosis, CSF CTC analysis may potentially also alter prognostication and treatment in LM. Unlike traditional CSF cytology, CSF CTC analysis provides a quantitative measure of tumor cells. As such, the CSF CTC enumeration holds the potential to serve as a biomarker. In patients with LM secondary to breast cancer receiving intrathecal trastuzumab, a decreased number of CTCs was reported in those responding to treatment.^22^ The study also reported increased CTCs in three patients approximately one month prior to clinical signs of progression.^22^ These early findings suggest CTCs could provide quantitative measures of response, recurrence, and progression. CSF CTCs may also have a prognostic significance: increased serum CTCs portend a worse prognosis in solid tumors^23^; changes in the number of CSF CTCs on serial sampling may similarly predict prognosis, providing essential information for risk stratification, and treatment planning.

Finally, CTC analysis allows for isolation of single tumor cells for DNA, RNA, and protein analysis.^23–25^ In a study of breast cancer patients with LM, genomic analysis of CSF CTCs identified alterations commonly found in primary breast tumors, confirming the breast tumor origin of the isolated cells.^26^ When CSF CTC DNA was compared to archival primary tumor DNA, clonal divergence was identified with alterations such as 8q24 gain more frequently observed in CSF CTCs compared to archival primary tumor tissue.^26^ The 8q24 gain includes the MYC locus, which represents a potential opportunity for future targeted treatment.^26^ In another study of eight patients with breast cancer and LM, the breast origin of isolated CTCs and evidence of clonal divergence were again noted.^24^ Drug sensitivity testing was performed on CSF CTCs with CDK4/6 inhibitor palbociclib identified as the agent with greatest antitumor effect. Similar results were obtained from a study of non-small cell lung cancer (NSCLC) patients with LM, which showed a high degree of concordance between primary tumor DNA and CSF CTC DNA, but also identified common NSCLC treatment resistance mutations such as amplification of the MET proto-oncogene and erythroblastic oncogene B2 (ERBB2) mutation.^25^ In a study of two patients with breast cancer and three patients with NSCLC-associated LM, single-cell RNA sequencing from CSF CTCs identified that cancer cells, but not CSF macrophages expressed iron-binding protein lipocalin-2.^27^ The study suggested cancer cells could outcompete macrophages for iron by lipocalin-2 expression, representing an important pathway for tumor cell proliferation within the CSF microenvironment.^27^ In mouse models, iron chelation therapy suppressed cancer cell growth, representing a potential treatment target.^27^

Together, these findings demonstrate proof-of-principle that CSF CTCs capture tumor heterogeneity present in LM. Study of CSF CTCs can identify mechanisms tumor cells utilize to survive and proliferate in CSF, lea. Comparison of primary tumor DNA to CSF CTC DNA will help elucidate mechanisms by which tumor cells adapt to and proliferate within the leptomeningeal space. Moreover, understanding the genomic profile of systemic as well as CSF tumor cells can assist with treatment planning, helping oncologists select agents with the greatest likelihood of success. CSF CTC analysis may also help identify resistance mutations, directing selection of further treatments and informing prognosis.

### Cell-free Tumor DNA

An additional emerging technology, cell-free tumor DNA (ctDNA) analysis, may aid in characterization of tumor heterogeneity in LM. DNA is released from neoplastic cells as well as healthy cells into the bloodstream, where it can be isolated and studied.^28^ Plasma ctDNA can be used to detect actionable mutations and resistance mechanisms that can aid in treatment planning.^28^ In a similar fashion, CSF ctDNA can provide detection of clinically relevant mutations from metastatic brain tumors as well as primary brain tumors.^29^

Early data employing for CSF ctDNA sequencing demonstrates the utility of this analysis: cancer residing with the CSF may harbor mutations distinct from those of the primary tumor^30,31^: In twenty-eight patients with LM secondary to NSCLC harboring epidermal growth factor receptor (EGFR) mutations, CSF ctDNA harbored unique mutations and copy number variations in CSF.^30^ As an example, MET copy number gain was most frequently identified in CSF ctDNA analysis of this patient population, followed by ERBB2, KRAS, ALK, and MYC.^30^ Loss of heterozygosity of TP53 was much more frequent (73.1%, 19/26 patients) in CSF ctDNA compared to plasma ctDNA (7.7%, 2/26 patients).^30^ EGFR T790M, a resistance mutation against tyrosine kinase inhibitors (TKIs) targeting EGFR-mutant tumors, was identified in CSF ctDNA of seven patients experiencing progression on TKI.^30^ Similarly, in a larger study of seventy-two patients with LM secondary to NSCLC, CSF more reliably identified NCSLC driver mutations when compared with plasma.^32^ EGFR mutations, ALK fusions, and ERBB2 amplifications were all more frequently observed in CSF compared to plasma.^32^ However, EGFR T790M was more frequently detected in plasma (15.3%, 11/72) compared to CSF (2.8%, 2/72).^32^ TP53 loss of heterozygosity was much more frequent in CSF ctDNA (41.7%, 30/72) compared to plasma (13.9%, 10/72).^32^ In a series of 11 patients with BRAF-driven malignancies, CSF ctDNA detected BRAF mutations in 3/3 patients with radiographic evidence of LM and 2/5 patients with brain parenchymal metastases.^31^ Conventional cytology was negative for tumor cells in one patient with radiographic evidence of LM while CSF ctDNA detected tumor-derived DNA.^31^

Beyond detection of tumor mutations, CSF ctDNA may have utility in LM detection and monitoring: In a case series of two patients with lung cancer, CSF ctDNA analysis revealed mutations in a signaling pathway oncogene K-ras prior to radiographic and cytologic confirmation of LM.^33^ Though a small series, further validation may demonstrate that ctDNA may provide diagnosis in the setting of negative cytology and imaging findings or identify individuals at risk for developing LM. Further, ctDNA may be used to assess treatment response or disease progression in the CSF.^31^ Whereas a traditional biopsy captures tumor makeup at a single timepoint, liquid biopsies allow longitudinal measurement of tumor characteristics.^28^ This may allow for the detection of escape mutations prior to accumulation of new neurologic deficits. Taken together, these findings illustrate the ability of CSF ctDNA analysis to identify driver mutations, detect presence of LM, capture and describe clonal divergence among populations of tumor cells, and potentially guide treatment planning by identifying resistance mutations or novel pathways for targeting. Additional advances in DNA extraction and analysis technology will likely further improve the yield and utility of ctDNA analysis.^34^ Prior to widespread clinical implementation, further validation of both CSF CTC and CSF ctDNA technologies are essential.^35^

## Therapeutic Advances in Leptomeningeal Disease

Current clinical practice is dominated by the poor outcome of LM. The primary goals are therefore symptomatic management by surgical and/or radiation-based therapies followed by intrathecal and/or systemic chemotherapy (Table 2).^4^ Due to the lack of large randomized controlled clinical trials, evidence-based treatment recommendations in LM are limited.^4^ In absence of prospectively validated criteria, assessment of treatment response can also be challenging. To address this issue, a Response Assessment in Neuro-Oncology (RANO) workgroup proposed criteria for LM response to treatment which combines neurologic examination, CSF analysis, and MRI findings.^36^ The original RANO proposal for response criteria provided a scorecard for radiographic assessment of LM with points assigned for presence of subarachnoid or ventricular nodules, leptomeningeal enhancement, and cranial nerve enhancement to characterize brain and spine involvement while noting parenchymal and epidural involvement.^36^ Due to discordant scoring noted upon review of MRIs from 22 patients with LM, a simplified scorecard noting the presence or absence of subarachnoid/ventricular nodules and leptomeningeal linear enhancement in the brain and the spine was proposed, but needs further validation.^36^ European Association of Neuro-Oncology (EANO) and European Society for Medical Oncology (ESMO) have jointly issued practice guidelines for diagnosis and treatment of LM.^37^ EANO-ESMO guidelines provide a treatment algorithm based on patient’s prognosis, presence of concurrent brain metastases, and state of extracranial disease.^37^

**Table 2.** Overview of Commonly Employed Treatments for Leptomeningeal Disease

Modality	Treatment	Overview
Surgery	Ventriculoperitoneal shunting	Symptomatic management of increased intracranial pressure
Radiation	Craniospinal radiation Whole brain radiation Focal radiation	Symptomatic relief and improvement in neurologic function, but no survival benefit
Intrathecal Chemotherapy	Methotrexate Cytarabine Thiotepa	Similar efficacy across single-agents; very limited data on multi-agent treatment
	Rituximab	60–76% response in patients with LM due to primary or secondary central nervous system lymphoma
	Trastuzumab	Possible efficacy in management of Her2+ breast cancer
Systemic Chemotherapy	High-dose IV methotrexate	Modest survival benefit
	Capecitabine	Case reports of efficacy in LM due to breast or esophageal cancer
	High-dose IV cytarabine	Limited efficacy in LM from solid organ tumors
	Temozolomide	Limited efficacy as single agent in LM from solid organ tumors
Targeted Chemotherapy	Dabrafenib Vemurafenib	Case reports suggesting efficacy in LM from BRAF V600E mutant melanoma naïve to BRAF inhibitor therapy
	Trametinib	Case reports suggesting efficacy in LM from BRAF V600E mutant melanoma naïve to MEK inhibitor therapy
	Osimertinib	Preclinical, phase I, and retrospective data suggesting efficacy in treating LM in patients with EGFR T790M-mutated NSCLC
	Alectinib Lorlatinib	Case reports suggesting efficacy in treating LM in patients with NSCLC with ALK chromosomal arrangement
	Bevacizumab	Addition to existing regimens in breast cancer and EGFR-driven NSCLC may improve CNS response in patients with LM

EGFR, epidermal growth factor receptor; LM, Leptomeningeal metastasis; NSCLC, non-small cell lung carcinoma; VEGF, vascular endothelial growth factor.

### Surgical Therapy

Surgical resection of metastatic lesions is not within the standard treatment protocol of LM. Neurosurgical intervention can be needed, however through the management of obstructive hydrocephalus and increased intracranial pressure. Treating hydrocephalus from LM can include high volume lumbar puncture for symptomatic relief and placement of ventriculoperitoneal shunt (VPS) systems if needed for longer-term relief.^38,39^ Despite short overall survival, VPS placement is an effective palliative measure^38,39^ with peritoneal carcinomatosis an extremely rare complication.^39^

### Radiation Therapy

Radiation therapy to symptomatic sites is standard palliative care for LM. Multiple RT modalities are available, including craniospinal RT, whole-brain radiation therapy (WBRT), or focal radiation (stereotactic or external beam) to areas of bulky disease.^40-43^ WBRT provides symptomatic relief and improves neurologic function, though it does not confer a survival benefit, particularly in the setting of breast and lung cancer.^44,45^ Expanding the treatment field further, (photon) craniospinal radiation is associated with significant myelosuppression, severely limiting its utility in patients receiving chemotherapy.^40^ To build on the palliative benefits of RT and limit toxicities, several investigators have turned to proton beam RT. Though prospective data and direct comparisons of proton versus photon radiation are lacking, use of proton RT may lead to lower radiation exposure decrease toxicities such as myelosuppression.^46^

### Intrathecal Chemotherapy

Chemotherapy may be delivered directly into the leptomeningeal space directly into the lumbar cistern via lumbar puncture or the intraventricular space using a surgically-placed catheter (Ommaya).^4^ This approach is reserved for patients with nonbulky disease and normal CSF flow dynamics. In retrospective studies, image-guided placement of intraventricular catheters has been shown to be accurate and safe,^47^ providing homogeneous distribution within the subarachnoid space.^48^ Retrospectively, patients receiving IT chemotherapy via intraventricular catheter demonstrated better overall survival than those receiving IT therapy to the lumbar cistern.^49^

Currently, methotrexate, cytarabine, and thiotepa are the most common agents administered intrathecally for LM secondary to solid tumors. However, optimal dosing and duration of treatment remains unclear and direct comparisons in clinical trials are lacking.^4,48^ Standard preparations of IT methotrexate, cytarabine, and thiotepa are all administered twice a week for four weeks, followed by four weeks of weekly infusions with once a month maintenance thereafter.^48^ Efficacy of these three single agents is similar.^48,50,51^ Very limited data is available for combination IT chemotherapy: In a study of fifty-five patients with LM, median survival was longer in patients treated with cytarabine and methotrexate combination therapy versus methotrexate alone, median survival was longer in the combination therapy group, but this has not been validated in larger prospective studies.^52^ Given singly, toxicities from IT methotrexate, thiotepa, and cytarabine administration are similar and include headache, nausea, vomiting, and fever, which are common sequelae of chemical meningitis and arachnoiditis^48,53,54^ as well as hemorrhage and infectious meningitis.^52,55^ IT methotrexate in particular, is associated with delayed leukoencephalopathy.^55^ The incidence chemical meningitis or arachnoiditis related to IT chemotherapy remains unknown. However, in one study, significant adverse events including paresthesias and paralysis were reported in 8.3% of patients undergoing IT chemotherapy whereas minor events such as headache, back pain, or nausea occurred in 26.6% with strong correlation between total number of IT treatments received and likelihood of at least one adverse event.^53^ Attempts to develop IT formulations from other untargeted IV agents such as topotecan or etoposide have been less effective.^56,57^

Select systemic antibodies may be delivered IT. Rituximab, a monoclonal antibody to CD20, delivered IT was associated with 60–76% response in patients LM due to primary or secondary central nervous system (CNS) lymphoma.^48,58^ Intrathecal checkpoint inhibitor administration has been attempted in LM due to melanoma^59^ and is the subject of clinical trial NCT03025256.^60^ The monoclonal antibody against Her2/neu, trastuzumab, has poor CNS penetration when given systemically, but given IT, trastuzumab has been well-tolerated and may have a role in management of Her2+ breast cancer LM.^61,62^ Similar repurposing of targeted treatment agents for melanoma and lung cancer may yield effective treatments against LM from solid organ tumors.

### Systemic Chemotherapy

Systemic chemotherapy is suitable for patients with bulky disease or impaired CSF flow. Moreover, this approach eliminates need for intraventricular catheter placement or repeated lumbar punctures.^4,63,64^ However, despite the rationality of this treatment approach, randomized trials are lacking; conventional systemic agents have limited efficacy in LM to date.^4,63^ High-dose IV methotrexate achieves cytotoxic concentrations within CSF, has been associated with modest survival benefit, and is among the most commonly used systemic agents for LM,^64^ particularly for LM due to breast cancer. Capecitabine has been employed in several case reports demonstrating efficacy in LM secondary to breast cancer^4,65^ or esophageal cancer.^66^ Other agents that have been considered for systemic chemotherapy of LM include cytarabine and temozolomide. High-dose IV cytarabine can achieve cytotoxic concentrations within CSF, but efficacy of this treatment in patients with LM from solid organ tumors has not been demonstrated.^67^ Commonly used in management of primary brain tumors, the alkylating agent temozolomide is not effective against LM as a single agent.^4,68^

### Immunotherapy

Immunotherapy with checkpoint inhibitors is under investigation for management of LM. In a single-arm phase II study, 17 patients with breast cancer, two with lung cancer, and one with ovarian cancer were treated with pembrolizumab.^69^ Primary endpoint of survival at three months was achieved with 12 of 20 patients alive after three months of enrollment, though median overall survival was 3.6 months.^69^ Response to nivolumab treatment in a patient with LM secondary to NSCLC and another with LM secondary to renal cell carcinoma have also been reported.^70,71^ These preliminary findings suggest there may be a role for checkpoint inhibitor therapy in management of LM, particularly in patients who have not received immunotherapy as part of their treatment regimen at initial diagnosis.

### Targeted Therapy

Overall, conventional cytotoxic chemotherapy has not demonstrated a prospectively validated clear sustained survival benefit in patients with LM. To date, no targeted chemotherapy has received Food and Drug Administration (FDA) approval for treatment of leptomeningeal disease. However, select targeted chemotherapies present novel and effective treatment strategies for LM and may be validated with further studies.

In patients with LM from melanoma harboring BRAF V600E mutations, there are reports of LM response to BRAF inhibitors such as dabrafenib and vemurafenib^72^ and the MEK inhibitor trametinib.^73^ Combined BRAF and MEK inhibitor therapy in BRAF-driven LM malignancies may be effective in patients naïve to these agents. However, in practice, patients harboring LM from melanoma have typically already been treated with BRAF and MEK inhibitors.^74^

In patients with EGFR-mutated NSCLC, tyrosine kinase inhibitors such as erlotinib and gefitinib are demonstrably effective first-line therapeutics.^75,76^ However, resistance mutations invariably develop. Third-generation TKIs such as osimertinib and rocetilinib have been designed to overcome the most common resistance mutation EGFR T790M.^77,78^ Osimertinib penetrates the CNS and appears effective in treating LM in patients with EGFR T790M-mutated NSCLC.^79^ Similarly, in patients with NSCLC with ALK chromosomal arrangements, the small molecule TKI crizotinib has been an effective first-line treatment.^80^ Tumor resistance to crizotinib has been recognized, leading to development of additional ALK inhibitors.^80^ Of these newer agents, alectinib demonstrates some efficacy against LM,^81^ there is anecdotal evidence of similar efficacy of lorlatinib in LM.

The vascular endothelial growth factor (VEGF) inhibitor bevacizumab also shows promise: High levels of VEGF are associated with LM diagnosis^82^ and CSF VEGF levels are negatively correlated with LM survival.^83^ Addition of bevacizumab to existing regimens improves CNS response in LM: In breast cancer-associated LM, combination therapy with bevacizumab, etoposide, and cisplatin (BEEP therapy) demonstrated CNS response and improved overall survival.^84^ Similar results have been reported in small case series of addition of bevacizumab to erlotinib in EGFR-driven NSCLC LM.^85^ Together, these reports suggest addition of bevacizumab to existing chemotherapy regimens may improve CNS response in patients with LM.

## Future Perspective

Recent advances in detection, quantification, and targeting of cancer cells within the spinal fluid have the potential to revolutionize the study and management of LM. Emerging CSF CTC and ctDNA technologies will improve diagnosis of LM and serve as biomarkers to assess disease progression and response to treatment, empowering intelligent clinical trial design and interpretation. CTC and ctDNA analysis will also provide a more detailed understanding of tumor genomics, paving the way to individualized targeted treatments focused on actionable mutations. In this way, liquid biopsies together with new targeted therapeutic approaches will enable formal clinical trials of LM enrolling carefully selected patients with quantitatively similar burden of disease, harboring biologically similar disease (as determined by driver mutation), receiving therapeutics targeting the driver mutation. This work will allow for establishment of treatment paradigms beyond our current regimens of symptomatic management with radiation therapy and ventriculoperitoneal shunting. With these tools in hand, we will make best use of intrathecal and systemic targeted and chemotherapeutic approaches to make clinically meaningful improvements for patients with LM.
